# Artificial Intelligence in Obstetrics: Current Trends and Future Directions

**DOI:** 10.3390/jcm15156059

**Published:** 2026-08-04

**Authors:** Ittai Many, Ariel Many

**Affiliations:** Gray Faculty of Medical and Health Sciences, Tel Aviv University, Tel Aviv 6997801, Israel

**Keywords:** artificial intelligence in obstetrics, fetal echocardiography, cardiotocography, labor prediction, ultrasound image analysis, external validation

## Abstract

**Background:** Artificial intelligence (AI), which spans machine learning (ML), deep learning (DL), computer vision, and natural language processing (NLP), is now used across obstetric care, including ultrasound interpretation (biometry, anomaly detection), fetal monitoring (cardiotocography), maternal risk stratification (preeclampsia, preterm birth, hemorrhage), labor and delivery decision support, genomic screening, and telehealth. **Methods:** We conducted a narrative (non-systematic) review of the literature published between 2016 and 2026, distinguishing the level of evidence supporting each application. **Results:** Reported performance is frequently high for image-based tasks such as fetal biometry and anomaly detection (accuracy and AUC often exceeding 0.85), whereas intrapartum CTG analysis remains modest (AUROC ~0.60–0.70, overlapping the inter-observer variability of clinicians). Most published evidence is retrospective and internally validated; comparatively few tools have undergone external or prospective validation, and only a small number have received regulatory clearance. **Limitations:** The evidence base is heterogeneous, external validation and calibration are often absent, and we did not perform a formal risk-of-bias appraisal. **Conclusions:** AI has real potential to improve prenatal diagnosis and individualized care, but claims that it is ready for the clinic are often premature. Prospective and external validation, calibration and clinical-utility assessment, transparent reporting, attention to bias and equity, and sustained clinician oversight are prerequisites for safe adoption.

## 1. Introduction

Artificial intelligence (AI) broadly encompasses machine learning (ML; traditional algorithms such as logistic regression and random forests), deep learning (DL; neural networks such as convolutional neural networks [CNNs] and recurrent neural networks [RNNs]), computer vision (image analysis), and natural language processing (NLP). These technologies are now used across obstetrics. ML and DL can automatically extract complex patterns from large datasets (images, signals, electronic health records [EHR]) to support diagnosis and prediction [1,2]. For example, AI algorithms have learned to identify subtle ultrasound features associated with congenital anomalies that may elude clinicians [3]. Key AI tools in obstetrics include CNNs for ultrasound image analysis, recurrent and attention-based models for time-series data (e.g., CTG signals), ensemble learners for risk prediction, and, more recently, large language models (LLMs) for information synthesis and patient communication. These technologies have been applied across the pregnancy continuum, from early screening and anomaly detection to intrapartum monitoring and postpartum risk assessment.

Emerging literature shows that AI systems can approach or exceed human-level performance in many obstetric tasks. For instance, a multicenter study demonstrated that DL models placed ultrasound biometry calipers with “human-level performance” in 20-week scans, providing calibrated confidence intervals for measurements [4]. Similarly, DL has achieved 96.3% accuracy classifying normal vs. abnormal fetal brain ultrasound [5]. In clinical screening, FDA-cleared AI tools have increased fetal congenital heart defect detection to >97% sensitivity, outperforming unaided expert review [6]. AI models have also generated delivery date predictions from routine sonograms with an R2 up to 0.92 [7,8]. These successes suggest AI’s potential to enhance diagnostic precision, standardize interpretation, and shorten workflow time in perinatal imaging.

However, many obstetric AI studies are retrospective or proof-of-concept in nature, and few have undergone rigorous prospective trials or independent external validation. Real-world deployment raises further challenges: performance can vary with data quality, patient demographics, and equipment. Ethical and regulatory frameworks are still evolving, and the limited explainability of many complex models, often called “black boxes”, holds back the trust that physicians and regulators need. Several narrative and scoping reviews have surveyed AI in obstetrics; the present review adds an explicitly critical, clinically oriented appraisal that weighs reported performance against study design, validation status, and readiness for real-world use. It is structured as a narrative (non-systematic) review and covers core technologies; applications (prenatal screening, ultrasound analysis, fetal monitoring/CTG, labor and delivery decision support, maternal risk stratification, genomics/NIPT and telehealth); and cross-cutting issues of validation, performance metrics, bias and fairness, explainability, clinical trials, regulatory approval, workflow integration, medicolegal and privacy considerations, and future research priorities.

## 2. Methods

This work is a narrative (non-systematic) review. We performed a structured but non-exhaustive search of PubMed, Embase and Google Scholar for studies published from January 2016 through March 2026 (with a small number of earlier landmark reports). Search terms combined “artificial intelligence”, “machine learning”, “deep learning”, “computer vision” and “natural language processing” with obstetric keywords (e.g., “prenatal screening”, “ultrasound”, “cardiotocography”, “preeclampsia”, “telemedicine”). We also examined relevant guidelines (WHO, ACOG, RCOG). We did not systematically search engineering databases such as IEEE Xplore, and relevant technical work indexed only there may have been missed. Peer-reviewed articles, systematic reviews, and major reports on AI in obstetric care were prioritized, with preference for higher-impact clinical and AI-in-medicine venues; non-English articles, opinion pieces without data, and studies unrelated to clinical obstetrics were excluded. Because this is a narrative review, we did not register a protocol, screen studies in duplicate, provide a PRISMA flow diagram, or perform a formal risk-of-bias appraisal (e.g., PROBAST or PROBAST-AI); reporting follows the transparency principles of PRISMA [9] where applicable, but the search is not intended to be exhaustive or fully reproducible, and no meta-analysis was performed. Throughout, we distinguish the level of evidence supporting each application (proof-of-concept, internal validation, external validation, prospective validation, randomized or implementation evidence, and regulatory clearance), because high discrimination in a retrospective, internally validated study should not be equated with clinical readiness.

### 2.1. Key AI Technologies

AI in obstetrics draws on several core technologies (Table 1):

Machine Learning (ML): Traditional ML methods (logistic regression, decision trees, random forests, support vector machines, and gradient-boosted trees such as XGBoost [extreme gradient boosting]) are widely used for risk modeling. Their strengths include interpretability and lower data needs, but they often plateau in complex pattern recognition. Many risk-prediction models (e.g., for preeclampsia or hemorrhage) use ML classifiers or ensemble methods [10,11].

Deep Learning (DL): DL—especially convolutional neural networks (CNNs)—excels in image analysis (e.g., ultrasound frames) and signal patterns. Recurrent neural networks (RNNs) and attention-based models are employed for time-series data (e.g., CTG tracing). DL models automatically learn features, often surpassing traditional ML in complex tasks; for example, CNNs achieved >90% accuracy in fetal anatomical image classification [5]. However, they require large datasets and are often “black boxes”—users know the input and the output, but the in-between process remains mostly non-explainable.

Computer Vision: Computer vision techniques analyze imaging data. In obstetrics, these methods detect standard ultrasound planes (e.g., head, abdomen), measure biometry (head circumference, femur length), and flag fetal anomalies. Examples include automated caliper placement (fetal biometry) [4] and segmentation of fetal structures. Advanced vision models (like U-Nets) enable real-time ultrasound guidance and volumetric measurements.

Natural Language Processing (NLP) and Large Language Models (LLMs): Although traditional NLP applications remain relatively under-explored in obstetrics, LLMs are advancing quickly and could be useful in clinical workflows. Recent models have performed well on complex medical material; for instance, one study reported that ChatGPT outperformed human candidates in postgraduate OBGYN examinations and produced diagnostic suggestions comparable to domain experts [12]. Beyond examination performance, plausible obstetric uses include drafting and summarizing clinical documentation, generating structured reports, patient education and triage, condensing guidelines, multilingual communication, and literature review. However, integrating these models into practice demands strict caution: the built-in risk of “hallucination”, the confident generation of wrong or fabricated information, creates severe safety risks in a field where a single wrong statement about a medication or warning sign can be catastrophic. Because these systems can be confidently wrong, they need firm safeguards, not just general caution: grounding outputs in verified sources through retrieval-augmented generation, careful prompt design, limiting use to lower-risk non-diagnostic tasks, and requiring human review of any patient-facing or decision-relevant output. To date, evidence for LLMs in obstetrics is largely limited to examination-style benchmarks, with little prospective clinical evaluation.

**Figure 1:**
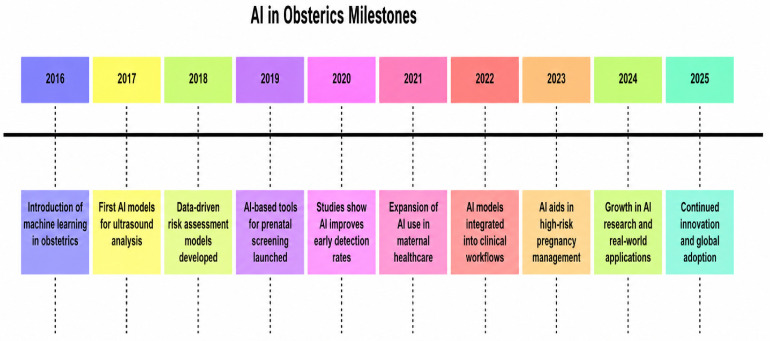
Timeline of major AI-related milestones in obstetrics (selected examples with references).

**Figure 2:**
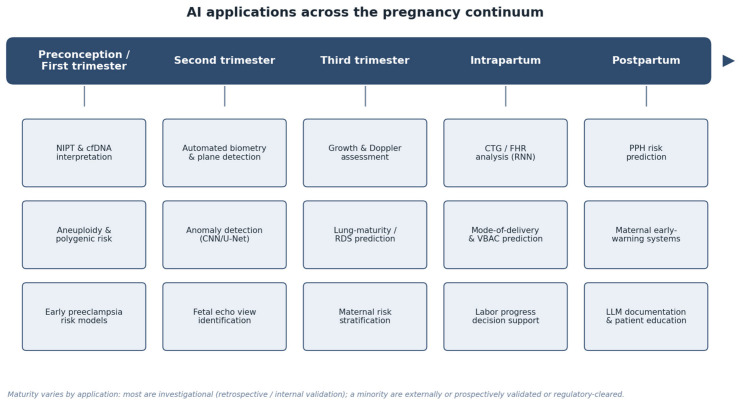
Artificial intelligence applications across the pregnancy continuum. Applications shown vary widely in maturity, from proof-of-concept to regulatory-cleared; most remain investigational.

These AI technologies can be applied to diverse obstetric data types (images, signals, text) to support decision-making. Figure 1 represents the history timeline of major AI-related milestones in obstetrics. Their integration should be guided by domain knowledge and clinical needs, as emphasized by guidelines recommending multidisciplinary collaboration and transparency [13]. In Figure 2 current Artificial intelligence applications across the pregnancy continuum are presented. Some are in routine use while others are investigational.

### 2.2. Applications of AI in Obstetrics

#### Prenatal Screening and Ultrasound Imaging

Fetal Anomaly Detection: Computer vision and DL have been used to detect structural anomalies from ultrasound. For example, Xie et al. (2020) developed a CNN-based pipeline to segment fetal brain structures and classify scans as normal or abnormal [5]. The model achieved 96.3% classification accuracy (sensitivity 96.9%, specificity 95.9%, AUC 0.989) [5]. Similarly, CNNs have been applied to first-trimester ultrasound frames to screen for limb, facial, and cardiac anomalies, often outperforming manual review. Commercial software (e.g., quantusFLM) can now analyze standard 2D images for fetal lung maturity, predicting neonatal respiratory distress with roughly 86–90% accuracy in multicenter trials [14]. These tools reduce operator dependence and can flag subtle findings for expert review.

Biometry and Growth Monitoring: AI models automate fetal measurements (e.g., head circumference, femur length). Venturini et al. trained a whole-examination CNN using 48 million video frames; its biometric estimates matched expert sonographers (“human-level performance”) and provided calibrated uncertainty intervals [4]. Portable ultrasound combined with AI (e.g., phone-based probes) has been validated in resource-limited settings: in rural Zambia, a mobile AI achieved GA estimates with mean error of ~±4.5 days versus standard ultrasound and accurately identified fetal presentation (AUC 0.977) [15]. AI-enabled ultrasound could democratize access, allowing midwives/nurses to perform reliable scans [16] and increase access to these technologies in rural areas.

Prenatal Screening (Genomic): AI is emerging in non-invasive prenatal testing (NIPT) and genomics. By analyzing cell-free fetal DNA sequencing data with ML, AI can improve detection of aneuploidies. One review notes AI can “help accurately predict Down syndrome” from maternal blood DNA patterns [17]. Polygenic risk scores for preterm birth or fetal conditions might also be refined by AI. Still, genomics-based AI is early-stage, with limited large-scale obstetric trials to date.

Fetal Echocardiography: AI assistance in fetal cardiac screening is proving effective. An FDA-approved deep learning tool now auto-identifies key cardiac views and anomalies in standard prenatal scans. Clinical use increased detection of critical congenital heart defects from baseline to >97% [6], reducing missed diagnoses. The AI also cut review time by ~18% and increased reader confidence [6].

### 2.3. Fetal Monitoring (Cardiotocography)

Intrapartum fetal heart rate (FHR) and uterine contraction monitoring (cardiotocography, CTG) have long suffered from subjective interpretation, and AI has been proposed as a route to more objective, standardized analysis [18]. DL models trained on CTG signals against outcomes (umbilical-artery pH, Apgar) have achieved only moderate success. For example, in one multicenter model-development study, a deep learning model trained on CTG signals paired with cord-blood pH reached an AUROC of only ~0.68 for detecting pH < 7.20, comparable to expert clinician interpretation [19]; models trained on objective pH labels tended to outperform those trained on subjective Apgar labels. More broadly, AI for CTG typically yields AUROCs in the 0.60–0.70 range [18,20], which beats chance but overlaps the inter-observer variability seen among expert clinicians and is well below the discrimination usually expected before a test should alter intrapartum management. These modest results reflect small and heterogeneous datasets, noisy biosignals, the absence of a uniform reference standard, subjective outcome definitions, and low reproducibility across centers, which together help explain why AI-based CTG has not entered routine practice. Explainable AI tools (e.g., saliency maps) are being introduced to highlight the features driving a decision, but AI-based CTG still needs to address population bias and to be validated prospectively [18,21]. From a delivery-suite perspective, the greater challenge is often not generating another score but acting on it: the most difficult decisions arise precisely with equivocal tracings, where an additional imperfect signal risks either unnecessary cesarean delivery or, through alert fatigue, desensitization to genuine warnings. Any intrapartum AI must therefore show that it improves decisions and outcomes, not merely classification metrics.

Besides CTG, novel sensors (e.g., fetal ECG) coupled with AI may offer future monitoring enhancements. Some proof-of-concept studies use ML on non-invasive ECG or Doppler signals for early distress prediction, though clinical translation is pending.

### 2.4. Labor and Delivery Prediction/Decision Support

AI can assist decisions around labor and delivery. For example, ML models have been trained to predict the likelihood of cesarean versus vaginal birth. A recent systematic review (17 studies) found that ensemble and neural models often achieved AUCs of ~0.90–0.93 in predicting mode of delivery (vaginal birth after cesarean [VBAC] success, emergency cesarean, etc.) [20]. Models that used real-time labor data (cervical dilation, contraction patterns) outperformed those using only baseline factors [20,22]; for instance, gradient-boosting models using partograph data reached AUC > 0.90 in one cohort [23]. In general, AI models substantially exceeded traditional logistic regression (often AUC ~0.6–0.7) when dynamic intrapartum data were used [22], although simpler models remained competitive when interpretability was prioritized. These figures, however, derive largely from retrospective, frequently single-center cohorts and usually report discrimination (AUC) without calibration, decision-curve analysis, or evidence that acting on the prediction improves outcomes; external and prospective validation remain scarce.

AI has also been applied to labor induction success, epidural response, and postpartum hemorrhage risk during delivery. For example, one predictive tool (XGBoost) using admission vital signs and history predicted PPH (blood loss ≥ 1000 mL) with C-index ≈ 0.93, outperforming clinician risk lists [11]. Similarly, early warning systems using ML on vital sign trends can flag deteriorating patients sooner than standard monitoring.

These decision support tools are currently investigational. Clinical trials embedding AI predictions into labor management pathways are few. Integration into practice will require demonstrating impact on outcomes (e.g., reduced unplanned C sections) without increasing interventions.

### 2.5. Maternal Risk Stratification

AI is now used to predict antenatal complications and maternal morbidity. The largest focus has been on preeclampsia: systematic reviews report ML models (random forests, XGBoost, elastic net, etc.) achieving AUCs of ~0.86–0.97 using first-trimester and routine data [10], although these estimates are heterogeneous and dominated by retrospective, internally validated studies. For example, a Taiwanese cohort (*n* = 2400) found that XGBoost predicted preeclampsia with an AUC of ≈0.92 using blood pressure, BMI, and routine laboratory results [24]. Similarly, ML has been applied to gestational diabetes, premature rupture of membranes, and other conditions, often leveraging routine antenatal records. In resource-rich settings, incorporating biomarkers (e.g., PAPP-A, placental growth factor) alongside clinical data can further improve reported model accuracy.

Other risk areas include postpartum hemorrhage (PPH): using a US registry of ~228,000 births, ML models (XGBoost, random forest) applied at admission predicted PPH (estimated blood loss ≥ 1000 mL) with a concordance index (C-index) of ≈0.92–0.95, better than logistic regression (~0.87) [11]. These models identified both known risk factors (placenta previa, macrosomia) and subtler interactions. Composite maternal morbidity (e.g., transfusion, ICU admission) is another target: ML applied to large EHR datasets can help flag high-risk patients at triage. Risk-stratification AI should support rather than replace clinical judgment, for instance by prompting preventive measures (aspirin, magnesium, closer follow-up) in high-risk women. As with other obstetric risk models, reported discrimination should be interpreted alongside calibration and demonstrable net clinical benefit, which are seldom reported.

#### Telemedicine and Resource-Limited Settings

AI can enhance remote obstetric care. During the COVID-19 pandemic, tele-OB/GYN usage surged over 500%, with AI-assisted triage cited as a key modality [25]. In low-resource areas lacking specialists, AI-driven mobile ultrasound allows trained non-experts to perform scans. The Clarius OB AI is FDA-cleared for handheld fetal biometry, explicitly aiming at rural/midwife settings [16]. By highlighting anatomy and auto-placing calipers, it reduces required expertise [16]. Similarly, AI-enabled tele-ultrasound (expert review of AI-annotated images) has improved access in remote clinics.

Other telehealth uses include AI chatbots for patient education and symptom triage. LLMs could power digital assistants for pregnant women, answering questions about medications or warning signs. For example, a chatbot was shown to handle OBGYN queries accurately (with oversight) and could help with medication reconciliation or lifestyle advice. However, tele AI must be used judiciously: barriers include electricity, internet access gaps (43% of rural Sub-Saharan clinics lack stable broadband), and digital literacy [26]. The telemedicine literature warns of equity issues; interventions must be designed to avoid widening disparities [27].

### 2.6. Data, Validation, and Performance Metrics

Datasets are the foundation of obstetric AI. However, public obstetric datasets are scarce and often small. Common sources include large clinical registries (e.g., NICHD Maternal Fetal Medicine Network data), public CTG archives (Oxford CTG database). Table 2 summarizes studies of artificial intelligence in obstetrics.

Training on multicenter data is important for generalizability, and external validation is needed. Several studies show that models often perform worse on external cohorts because of population or equipment differences; for example, CTG models exhibit a performance drop when applied at a different hospital [28]. Such dataset shift is driven by differences in patient demographics and case mix, ultrasound machines and acquisition protocols, operator technique, ethnic diversity, and the level of the healthcare facility, so a model that does well internally can lose accuracy in a new setting. To mitigate this, federated learning, in which models train locally and share only parameters, is being explored: in reproductive medicine, a federated model outperformed hospital-specific models while preserving patient privacy [29]. Similar approaches could allow obstetric data to be shared across hospitals without exchanging raw data, potentially improving performance and equity.

Validation of AI tools must be rigorous. Key metrics include the area under the ROC curve (AUC/AUROC), accuracy, sensitivity and specificity, F1 score, positive predictive value, and, for continuous outcomes such as gestational age, the coefficient of determination (R^2^). Discrimination alone, however, is insufficient. Common but under-reported pitfalls include data leakage, class imbalance, and poor data partitioning. One example is splitting ultrasound frames at the image rather than the patient level, which lets frames from the same fetus land in both the training and test sets and inflates apparent performance. Beyond discrimination, studies should report calibration (agreement between predicted and observed risk), clinical utility (e.g., decision-curve analysis), and performance within relevant subgroups. Every study should use separate test sets and, preferably, external validation to guard against overfitting; in practice, many models report cross-validated AUCs (e.g., ≈0.92 for XGBoost in preeclampsia [24]) but lack calibration, external validation, and independent prospective testing.

Comparisons with clinician performance or established tools are instructive. For instance, AI fetal biometrics were compared to sonographer measurements [4]; AI CTG models are often benchmarked against expert review. In many cases, AI outperforms baseline logistic models by a clear margin. For example, ML for PPH achieved AUC ~0.93 vs. logistic 0.87 [11], and delivery mode AI reached ~0.93 vs. ~0.75 for basic risk scores [20].

### 2.7. Bias, Explainability, and Ethics

AI systems must be scrutinized for bias. Training data often underrepresents certain groups (e.g., minority ethnicities, extreme BMI), risking skewed performance. For instance, if an AI CTG model is trained on data mostly from one population, it may not generalize to others. Guidelines explicitly call for “mitigating algorithmic bias” and transparent communication with patients [13]. Explainability tools (SHapley Additive exPlanations [SHAP] values, saliency maps) are increasingly used. For example, SHAP analysis in XGBoost preeclampsia models identified top predictors (blood pressure, glucose) and can reveal when a model over-relies on confounders [30].

However, explainability has limits: complex DL models remain opaque. Regulatory bodies and ethicists stress human oversight. A recent perspective warns that AI risk labels (e.g., “high-risk pregnancy”) can cause anxiety or stigma if not communicated properly [31,32]. There is a risk of reinforcing disparities: for example, “over-flagging socially disadvantaged groups” can occur if historical biases in care or data are encoded in AI [31]. Therefore, ethics frameworks for obstetric AI emphasize fairness audits, subgroup calibration, and combining risk scores with patient counseling [31,32].

Data privacy is another major concern. Prenatal data are sensitive (genomics, images, EHR). Regulations like HIPAA (US) and GDPR (EU) apply. AI development should incorporate privacy safeguards: de-identification, secure data storage, and, when possible, privacy-preserving techniques (federated learning [29], differential privacy). A scoping review noted that fragmented policies and unclear data governance are barriers to AI uptake in OB/GYN telehealth [33]. Ensuring patient consent and transparency about AI use are ethical imperatives.

Finally, patient autonomy and clinician accountability must be maintained: AI should support, not replace, obstetric judgment. Liability for AI-assisted decisions is still a legal gray area; current guidance suggests treating AI as decision support rather than an autonomous actor, but many questions are unresolved. It is often unclear where responsibility lies when a clinician overrides a correct AI alert, or follows an incorrect one, and how AI outputs will be treated as evidence in malpractice litigation. These worries are sharper in obstetrics, where one decision affects both mother and fetus. Providers must therefore verify AI recommendations, and institutions need clear policies on oversight, documentation and disclosure. Ongoing ethics research calls for interdisciplinary input (clinicians, data scientists, ethicists and patient advocates) in the design and deployment of these tools.

### 2.8. Clinical Trials and Regulatory Approval

Most AI in obstetrics remains at the research stage; few have formal regulatory clearance (Table 3). Notable exceptions are those integrated into medical devices:

FDA-cleared tools: the FDA has begun authorizing AI devices for obstetric use. In 2024, Clarius received clearance for its OB AI (handheld ultrasound for fetal measurements) [16]. In 2025, an AI system for fetal echocardiography (Cinc’s DxOne Fetal™) was FDA-authorized to aid detection of heart defects [6]. In early 2026, a “Delivery Date AI” system received De Novo authorization for predicting delivery timing from ultrasound [8]. These authorizations proceed largely under the Software as a Medical Device (SaMD) framework and often rest on demonstrating analytical or software performance, or substantial equivalence to a predicate device, rather than improved clinical outcomes; regulatory clearance should therefore not be read as proof of clinical benefit. AI-specific regulatory challenges include adaptive or continuously learning algorithms (addressed in part through predetermined change-control plans), requirements for transparency and labeling, and thorough post-market surveillance to detect performance drift once a tool is deployed at scale.

European approval: many AI ultrasound aids are CE-marked (via MDR or, for in vitro diagnostics, IVDR compliance), such as GE’s AI-based fetal echocardiography assist tool or Samsung’s automated biometry. CE marking does not, however, guarantee all performance claims, and clinicians must still rely on independent published evaluations. In addition, the EU Artificial Intelligence Act classifies many clinical decision-support systems as “high-risk”, imposing further obligations for risk management, data governance, transparency and human oversight that will increasingly shape how obstetric AI tools are developed and marketed.

Clinical trials: Few prospective trials of AI in OB have been completed. One example (ongoing) is the ‘PERFORM’ study comparing LLMs to residents (abstract only, no results yet) [34]. Most evidence is retrospective or cross-sectional. There is a pressing need for randomized or prospective implementation studies to assess impact on outcomes (e.g., does AI screening reduce missed diagnoses or unnecessary interventions?). Any AI incorporated into clinical care should ideally undergo phased trials, starting with pilot feasibility and moving to larger effectiveness trials. Prospective evaluation is especially difficult in obstetrics: randomizing an intervention that simultaneously affects mother and fetus raises particular ethical challenges, meaningful endpoints such as neurodevelopmental outcome may take years to ascertain, and a model that performs well in one setting can degrade when the population or ultrasound equipment changes. These barriers, rather than a lack of algorithms, largely explain why so little high-level prospective evidence yet exists.

Guidelines and standards: Professional bodies have not yet issued OB-specific AI guidelines (as of 2026), but general digital health frameworks apply. Research adherence to TRIPOD (for prediction models) and CONSORT-AI/DECIDE-AI (for clinical trials) is increasingly expected. Reporting standards (e.g., clearly stating data sources, validation methods) are critical to ensure reproducibility.

### 2.9. Integration into Clinical Workflows

Integrating AI tools into everyday obstetric care requires workflow compatibility and user training. For example, AI-assisted ultrasound often presents as an optional overlay on existing ultrasound machines or smartphone apps. Studies report that providers can adapt to AI guidance with brief training, which can also serve as a teaching aid [16]. AI suggestions (e.g., boundaries for fetal structures) should be easily toggled so clinicians retain control.

In fetal monitoring, AI outputs should integrate with electronic fetal monitoring (EFM) consoles or EHR alerts. User interface design (clear risk scores, trend graphs) influences acceptance. Studies have found clinicians prefer explainable outputs and the ability to review underlying signals.

Implementation also hinges on IT infrastructure (data connectivity, compute power) and interoperability (AI outputs feeding into hospital systems). Pilot implementations should involve end users to refine user experience and minimize “alert fatigue”. Cost–benefit analyses and reimbursement models also need consideration, e.g., will insurers cover AI-based scans or triage? And to what extent? Early evidence suggests improved diagnostic yield (e.g., higher CHD detection) could justify costs. Because obstetric clinicians already contend with heavy electronic-documentation burdens, tools that add clicks or generate frequent low-value alerts are unlikely to be adopted however accurate they are; minimizing added workload and alarm burden is therefore as important as predictive performance.

Training and changing management attitude are key. Surveys show some obstetricians are skeptical of “black-box” AI [1]. Education on AI capabilities and limitations for clinicians and patients is important. ACOG and RCOG have called for continuing education on emerging technologies. Ultimately, successful integration will require demonstrating that AI tools improve patient outcomes or efficiency without disrupting care.

### 2.10. Limitations of This Review

This review has several limitations. It is a narrative, non-systematic synthesis: the search was not exhaustive, engineering databases such as IEEE Xplore were not systematically searched, study selection was not performed in duplicate, and no formal risk-of-bias or quality appraisal (e.g., PROBAST or PROBAST-AI) was undertaken. The performance figures cited are drawn from heterogeneous studies with differing designs, populations and outcome definitions and are therefore not directly comparable, and several regulatory or product claims derive from manufacturer communications rather than peer-reviewed evidence. Accordingly, the summaries here should be read as a map of the field and its maturity rather than as a graded, quantitative assessment of individual tools (Table 4).

### 2.11. Future Research Directions

To fully realize AI’s potential in obstetrics, future work should address current limitations and explore new frontiers:

Data Sharing and Standards: The field needs large, diverse, high-quality datasets. Collaboration between national and international institutions (potentially via federated learning [29]) can aggregate data while preserving privacy. Standardization of data formats (e.g., DICOM for imaging, unified partographs) will help. Initiatives to create publicly available obstetric AI benchmarks (analogous to ImageNet in vision) could accelerate progress and enhance adoption.

Prospective Validation: Well-designed prospective and multicenter trials are needed. These should test AI tools in real clinical settings, measure health outcomes (e.g., detection rates, perinatal mortality), and monitor unintended effects. Regulators and the public are likely to demand such evidence.

Explainable and Multimodal AI: Advances in interpretable AI (e.g., prototype learning, attention maps) may build trust. Likewise, multimodal models that combine imaging, signals, EHR data, and even genomics could improve predictions (e.g., integrating ultrasound and lab values for growth restriction). Research into LLMs for clinical support (beyond exam performance [12]) could enable smarter EMR query and patient communication tools.

Addressing Bias and Equity: Future models must be audited for fairness across race, socioeconomic, and geographic groups. Techniques like subgroup calibration and bias mitigation need validation. Research on AI’s impact on health equity (does it reduce or worsen disparities?) is needed.

Regulatory and Ethical Frameworks: As AI becomes mainstream, clear guidelines are needed. Research into legal liability, consent models, and ethical AI use in pregnancy (e.g., limits on prenatal risk screening) should inform policy. Longitudinal studies on patient attitudes and psychosocial effects of AI (as warned by Ji et al. [31]) will be important.

Emerging Tech: The convergence of AI with other technologies (wearables, AR/VR, robotics) will shape the future. For example, AI-guided robotics for precise ultrasound probe placement or automated fetal resuscitation support is conceivable. Immersive simulations (digital twins of mother–fetus systems) are being explored for training.

Cost-effectiveness: Ultimately, health economic analyses should compare AI-driven care pathways to standard care to justify adoption.

In summary, the medical community is conservative and sometimes reluctant to new modalities. Nevertheless, AI is being implemented in various fields. Ongoing interdisciplinary collaboration will be important. Obstetricians, midwives, data scientists, ethicists, and patients must work together to ensure AI innovations are safe, effective, and aligned with women’s needs.

## Figures and Tables

**Table 1:** Comparison of core artificial intelligence approaches used in obstetrics.

AI Approach	Typical Obstetric Use	Data Needs	Interpretability	Clinical Maturity
Classical ML (logistic regression, random forest, XGBoost)	Risk prediction (preeclampsia, PPH, mode of delivery)	Low–moderate; tabular/EHR data	Moderate–high	Mostly investigational; some internally/externally validated models
Deep learning—CNNs	Ultrasound image classification, biometry, anomaly detection	High; large labeled image sets	Low (“black box”)	Some FDA-cleared imaging tools
Deep learning—RNN/attention	Time-series signals (CTG, FHR)	Moderate–high; labeled signal data	Low	Proof-of-concept to early validation
Computer vision (segmentation, U-Net)	Plane detection, caliper placement, structure segmentation	High; annotated images	Low–moderate	Embedded in some cleared devices
NLP/LLMs	Documentation, patient education, guideline/EHR summarization	Very high; large text corpora	Low	Early; largely benchmark evidence

**Table 2:** Representative studies of artificial intelligence in obstetrics discussed in this review.

Application	Study [Ref]	Design/Data	Model	Reported Performance	Validation
Fetal biometry	Venturini et al. [4]	Multicenter; ~48 M video frames, 20-wk scans	CNN (whole-examination)	Expert-level biometry with calibrated intervals	Internal (multicenter)
Fetal brain anomaly	Xie et al. [5]	Retrospective ultrasound images	CNN	Accuracy 96.3%, AUC 0.989	Internal
Congenital heart defect	Arnaout et al. [6]	Retrospective prenatal scans	Ensemble neural networks	Expert-level; >97% detection in reported clinical use	Internal; basis for cleared tool
Gestational age (low-resource)	Pokaprakarn et al. [15]	Prospective, rural Zambia; blind sweeps	DL	Mean GA error ~±4.5 d; presentation AUC 0.977	Field/external
Neonatal respiratory morbidity	Palacio et al. [14]	Multicenter; 2D lung-texture	ML (quantusFLM)	~86–90% accuracy	Multicenter
CTG/fetal hypoxia	Ref [19]	Multicenter model development	DL	AUROC ~0.68 (pH < 7.20)	Internal/AI-human comparison
Mode of delivery	Elhabeeb et al. [20]	Systematic review (17 studies), mostly retrospective	Ensemble/neural	AUCs up to ~0.90–0.93	Mostly internal
VBAC/vaginal delivery	Guedalia, Lipschuetz [22,23]	Retrospective cohorts; real-time labor data	ML/gradient boosting	AUC > 0.90 with intrapartum data	Internal
Preeclampsia	Shyu et al. [24]	Retrospective cohort (*n* = 2400)	XGBoost	AUC ≈ 0.92	Internal (cross-validated)
Postpartum hemorrhage	Venkatesh et al. [11]	US registry, ~228,000 births	XGBoost/RF	C-index ≈ 0.92–0.95	Internal

Performance figures are as reported by the original studies; most reflect internal or retrospective validation, use differing outcome definitions, and are not directly comparable. External or prospective validation is noted where available. AUC/AUROC, area under the receiver-operating-characteristic curve; GA, gestational age; PPH, postpartum hemorrhage; RF, random forest; VBAC, vaginal birth after cesarean.

**Table 3:** Selected regulatory-cleared and CE-marked AI tools in obstetrics.

Tool/Developer	Function	Regulatory Status (as Cited)
Clarius OB AI [16]	Handheld/portable fetal biometry	FDA-cleared (2024)
DxOne Fetal/Cinc [6]	Fetal echocardiography view and anomaly identification	FDA-authorized (2025)
Delivery Date AI/Ultrasound AI [8]	Delivery timing prediction from ultrasound	FDA De Novo (2026)
GE, Samsung ultrasound AI	Fetal-echo assist/automated biometry	CE-marked (MDR/IVDR)

Regulatory clearance reflects demonstrated safety and/or software performance, or substantial equivalence to a predicate device, and does not by itself establish improved clinical outcomes. Some entries are supported by manufacturer press releases [8,16] rather than peer-reviewed evidence.

**Table 4:** Current limitations of AI in obstetrics and corresponding priorities for future work.

Area	Current Limitation	Priority for Future Work
Evidence base	Most studies are retrospective, single-center, and internally validated.	Prospective, multicenter external validation and randomized or implementation trials.
Generalizability	Accuracy falls across new populations, ultrasound machines, operators, and sites (dataset shift).	Diverse, representative training data; site-specific recalibration; ongoing drift monitoring.
Reporting quality	Frequent data leakage, class imbalance, image- rather than patient-level splitting, and missing calibration.	Standardized reporting of calibration, decision-curve analysis and subgroup performance; risk-of-bias appraisal (e.g., PROBAST-AI).
Fetal monitoring (CTG)	Moderate AUROCs that overlap inter-observer variability; noisy signals; no uniform reference standard.	Better reference standards and larger datasets; proof that outputs change decisions and outcomes, not only metrics.
Explainability	Many high-performing models are opaque “black boxes”.	Interpretable outputs (e.g., SHAP, saliency maps) and interfaces clinicians can question.
Large language models	Risk of hallucination; evidence limited to examination-style benchmarks.	Retrieval-augmented generation, prompt design, use limited to lower-risk tasks, mandatory human review, and prospective clinical study.
Regulation	Clearance often rests on substantial equivalence or software performance, not clinical benefit; adaptive models are hard to govern.	Post-market surveillance, predetermined change-control plans and transparency; alignment with FDA SaMD, EU MDR/IVDR and the EU AI Act.
Medicolegal and ethics	Unclear liability when a clinician overrides or follows an AI output; consent and autonomy under-addressed.	Clear accountability frameworks that treat AI as decision support; guidance on informed consent and liability.
Workflow	Alert fatigue, added documentation burden and clicks, limited interoperability, unclear cost and reimbursement.	Human-centered design, smooth EHR integration, cost-effectiveness evidence and reimbursement pathways.
Equity	Risk of worsening disparities; under-representation of some groups in training data.	Bias audits, mandatory subgroup reporting and inclusive, representative datasets.

This table summarizes limitations and priorities discussed in the text and is intended as a narrative synthesis, not a formal quality assessment.

## Data Availability

No new data were created or analyzed in this study. Data sharing is not applicable to this article.
